# Multimodal imaging findings of primary liver clear cell carcinoma: a case presentation

**DOI:** 10.3389/fmed.2024.1408967

**Published:** 2024-05-16

**Authors:** Xianwen Hu, Xiaotian Li, Wei Zhao, Jiong Cai, Pan Wang

**Affiliations:** ^1^Department of Nuclear Medicine, Affiliated Hospital of Zunyi Medical University, Zunyi, China; ^2^Department of Pathology, Affiliated Hospital of Zunyi Medical University, Zunyi, China

**Keywords:** liver, clear cell carcinoma, imaging findings, MRI, PET/CT

## Abstract

Primary clear cell carcinoma of liver (PCCCL) is a special and relatively rare subtype of hepatocellular carcinoma (HCC), which is more common in people over 50 years of age, with a preference for men and a history of hepatitis B or C and/or cirrhosis. Herein, we present a case of a 60-year-old woman who came to our hospital for medical help with right upper abdominal pain. The imaging examination showed a low-density mass in the right lobe of his liver. In contrast enhanced computed tomography (CT) or T1-weighted imaging, significant enhancement can appear around the tumor during the arterial phase, and over time, the degree of enhancement of the tumor gradually decreases. The lession showed obviously increased fluorine-18 fluorodeoxyglucose (^18^F-FDG) uptake on positron emission tomography/CT. These imaging findings contribute to the diagnosis of PCCCL and differentiate it from other types of liver tumors.

## Introduction

Primary clear cell carcinoma of liver (PCCCL) is a relatively rare subtype of hepatocellular carcinoma (HCC) in histology, with clear cells accounting for 50% or more of the tumor and an incidence rate of approximately 0.9 to 8.8% of liver cancer (1, 2). The pathogenesis of PCCCL is not well understood. One study believed that its pathogenesis may be caused by the decrease of blood supply to the portal static vein, relative ischemia of the tumor, and the subsequent lipid and glycogen turbulence, and the organelles in the cytoplasm are replaced by glycogen and lipids, making the tumor cells appear clear or vacuolar (3). It is more common in more than 50 years of age, preferring men, most of them have a history of hepatitis B or C and/or cirrhosis, the patient has no characteristic clinical symptoms, mostly due to right upper abdominal pain or physical examination found liver occupation (4). PCCCL is a low-grade malignant tumor, mostly well differentiated, and often has a pseudocapsule, making it easier to undergo complete surgical resection. Its prognosis and survival rate are superior to other types of liver carcinoma, so obtaining a correct diagnosis before surgery is crucial (5). Herein, we present the diagnosis and treatment of a patient with primary PCCCL, focusing on its multimodal imaging features including computed tomorgraphy (CT), magnetic resonance imaging (MRI), and positron emission tomography (PET)/CT, with a view to increasing awareness of this rare disease.

## Case presentation

A 60-year-old woman had dull pain in the right upper abdomen without obvious reasons a month ago, no radiating pain in the shoulder and back, each pain lasting from 30 min to 1 h, 1–2 times a day, and the frequency increased in the past week, during which no treatment was performed. Ultrasound from an external hospital indicates a liver occupying lesion, and the patient came to our hospital for further diagnosis and treatment on February 3, 2024. Physical examination revealed tenderness in the right upper abdomen without significant rebound pain or other positive signs. The patient denied any history of hepatitis or other liver problems, and she and her family denied any history of cancer or genetic problems. The results of laboratory examination indicated that the liver function was impaired (the parameters of abnormal liver function are shown in Table 1) while the renal function index was within the normal reference value range. The results of serum tumor markers revealed an increase in carbohydrate antigen-199 (Ca-199) and alpha fetoprotein (AFP), with values of 541 U/mL (normal: 0–30 U/mL) and 66.9 g/L (normal: 0–20 ug/L), respectively. The values of other gastrointestinal tumor markers were within the normal range.

**Table 1:** Abnormal indixes of the patient’s liver function.

Indexes	Results	Unit	Annotation	Reference
Alanine aminotransferase	95.0	U/L	up	7–40
Aspartate aminotransferase	41.0	U/L	up	13–35
Glutamyltransferase	186.0	U/L	up	7–45
Total bilirubin	21.1	μmol/L	up	5–21
Direct bilirubin	9.0	μmol/L	up	0–3.4
Albumin	27.8	g/L	down	40–55
Globulin	47.0	g/L	down	20–40
Albumin/Globulin	0.6		down	1.2–2.4

**Figure 1:**
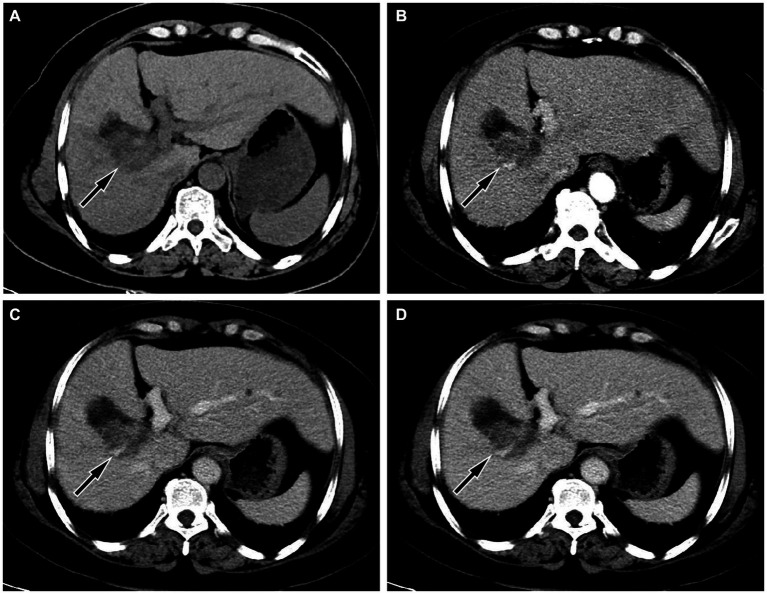
**(A)** Abdominal computed tomography (CT) scan showed a circular low-density shadow near the gallbladder fossa in the right lobe of the liver (arrow); moreover, the volume of the left lobe of the liver increases, the ratio of the left and right lobes is unbalanced, and the widening of the liver cleft can also be seen, suggesting cirrhosis; **(B)** In the arterial phase of contrast-enhanced CT scan, the lesion showed uneven enhancement, mainly with peripheral enhancement of the lesion (arrow); During the portal vein phase **(C)** and delayed phase **(D)**, the degree of enhancement of the lesion gradually declines (arrows).

**Figure 2:**
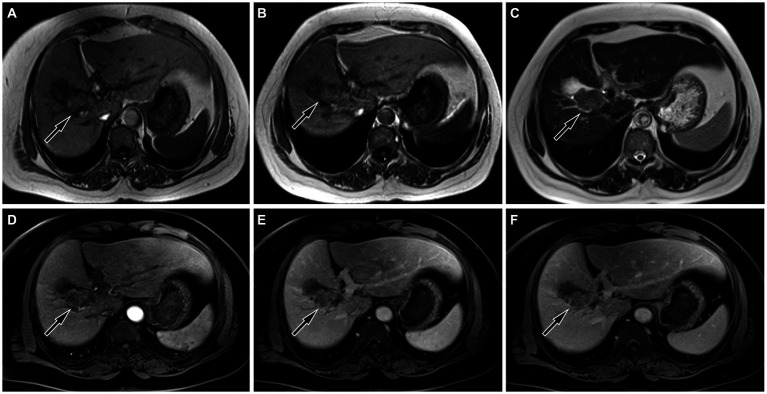
On magnetic resonance imaging (MRI), the lesion showed equal signal on both in-phase **(A)** and out-phase **(B)** of T1-weighted imaging (T1WI, arrows), and uneven slightly higher signal on T2WI (**(C)**, arrow). On contrast-enhanced T1WI, the lesion showed mild to moderate enhancement in arterial phase (**(D)**, arrow), portal phase (**(E)**, arrow) and delayed phase (**(F)**, arrow), mainly peripheral enhancement.

**Figure 3:**
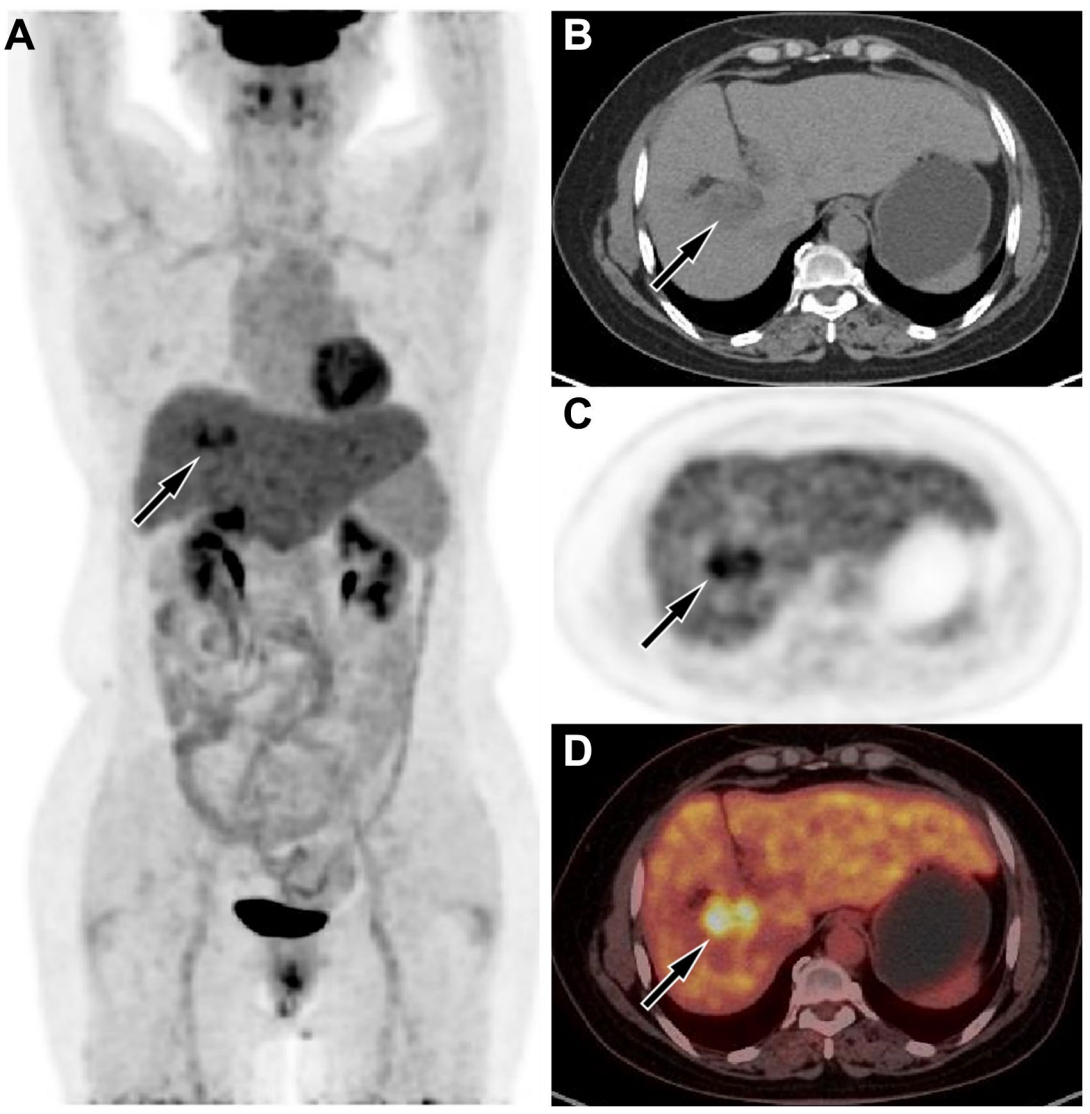
**(A)** The maximum intensity projection of the positron emission tomography (PET)/computed tomography (CT) showed a lesion in the liver region with increased uptake of fluorine-18 fluorodeoxyglucose (^18^F-FDG), with a maximum standardized uptake value (SUVmax) of 8.5. Axial CT **(B)** showed the lesion in the right lobe of the liver (arrow). Axial PET **(C)** and PET/CT fusion image **(D)** showed obviously increased ^18^F-FDG uptake of the mass (arrows).

**Figure 4:**
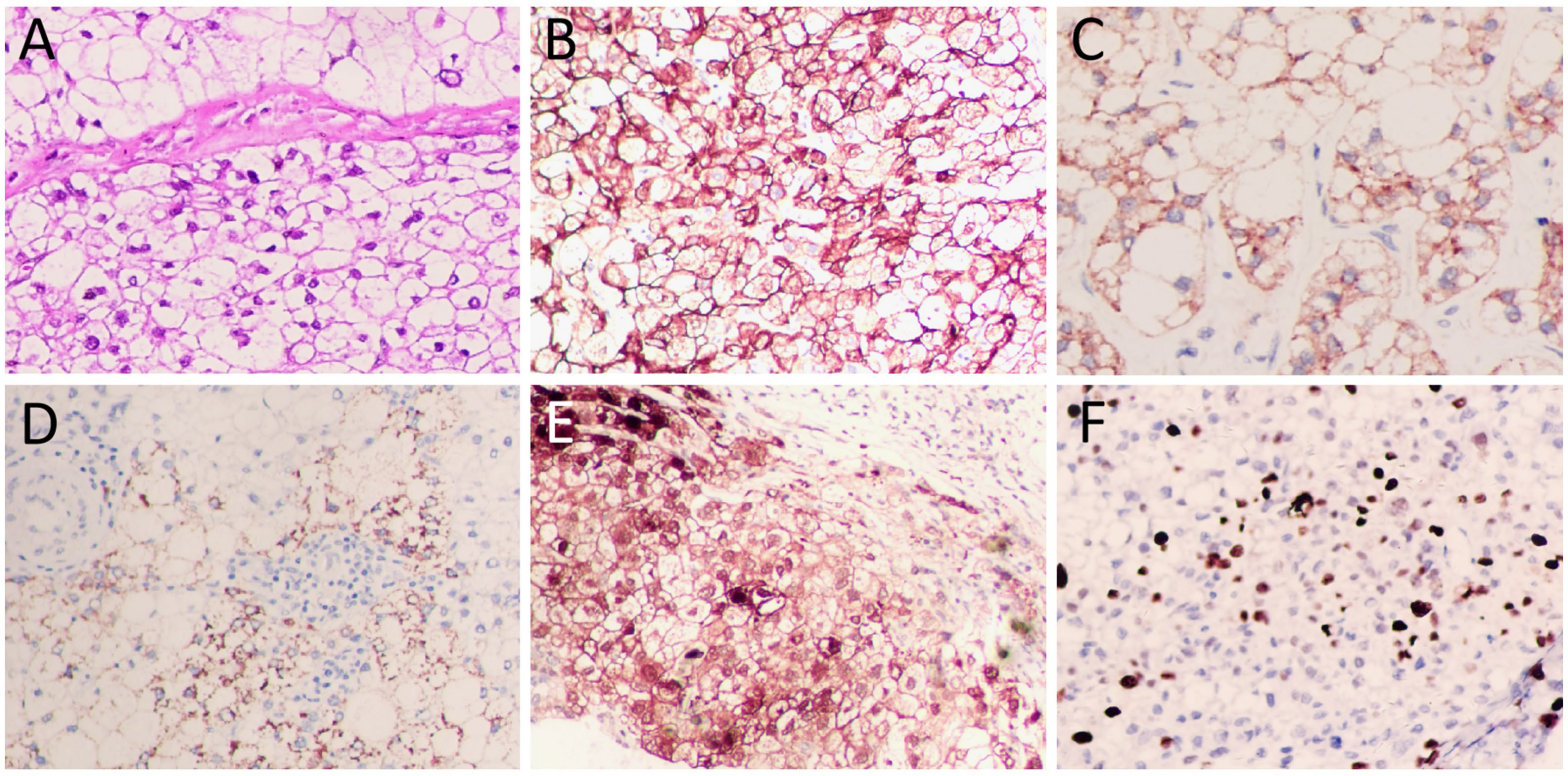
**(A)** Hematoxylin–eosin staining showed diffuse distribution of clear cells with abundant cytoplasm, centrally located nuclei and deep staining. Immunohistochemistry showed positive expression of tumor cells CK **(B)**, Hep par1 **(C)**, and Glypican (GPC3, **(D)**), HSP70 (partially, **(E)**), and Ki67 (about 40%, **(F)**). All images are 200 × magnification.

The upper abdominal CT scan (CT examination was performed using 16-detector-row scanners [Siemens, Germany] from the top of the liver to the lower pole of the kidney; The contrast-enhanced scan was performed with iohexol [300 mg I/mL]1.5 mL/kg, and a single phase injection at a rate of 3.0 mL/s was performed with a high pressure syringe) showed a circular low-density lession near the gallbladder fossa in the right lobe of the liver, with uneven mild enhancement on contrast-enhanced scanning (as shown in Figure 1); Moreover, CT showed that the left and right lobes of the liver were disproportioned, the left lobe was enlarged, and the liver fissure was widened, suggesting possible cirrhosis. The MRI (GE 3.0 T Signa HDxt superconducting magnetic resonance instrument, with abdominal coil; Conventional T1WI and T2WI sequence plain scan and contrast-enhanced scan were performed; and the contrast-enhanced scan was intravenously injected with 0.1mmoL/kg meglumine gadopenate, and the injection flow rate was 3.0 mL/s) of the upper abdomen showed that the lesion presented equal signal on T1WI and slightly uneven high signal on T2WI (Figure 2). Based on these imaging findings, the patient was initially suspected to have intrahepatic cholangiocarcinoma. In order to further evaluate the nature of the tumor and determine the optimal treatment plan for the patient, she underwent fluorine-18 fluorodeoxyglucose (^18^F-FDG) PET/CT examination (Biograph mCT, Siemens, Germany; The injection dose of ^18^F-FDG was 9.0 mCi [0.15 mCi/kg]), and the results revealed increased ^18^F-FDG uptake in the lesion, while no other increased radioactive uptake lessions were observed in the rest of body (Figure 3). After a thorough evaluation of the patient’s condition by the clinician, she underwent “total hepatectomy and allogeneic orthotopic liver transplantation” under general anesthesia on February 13, 2024. Postoperatively, the excised liver tissue will be sent for pathological examination. Under the microscope, the surface of the liver was miliary nodules, and a mass was found in the right lobe of the liver near the gallbladder, about 55 × 30 × 20 mm. The section was grayish-yellow solid, medium in quality, and the boundary between the surrounding tissues was clear. Hematoxylin–eosin staining showed that most of the lesions were clear cells with rich and clear cytoplasm, and the nucleus was located in the center, deeply stained and irregular (Figure 4). The immunohistochemical results showed that the tumor cells expressed hepatocyte paraffin 1 (Hep par1), CK, Glypican (GPC3), HSP70 (partially), and Ki67 (about 40%) positively, while AFP, CD10, and CD68 were negatively expressed. Based on these findings, the patient was ultimately diagnosed with PCCCL. After liver transplantation, the patient underwent contrast-enhanced CT scan of the upper abdomen, which showed rich liver blood supply, indicating successful surgery. The patient did not complain of any discomfort during follow-up up to now. A detailed summary of the patient’s journey is shown in Table 2.

**Table 2:** The outline of the patient journey.

Time flow	Affair	Results
02/01/2024	The patient had dull right upper abdominal pain without obvious cause	No processing is performed
01/02/2024	Abdominal ultrasound was performed in an outside hospital	A space-occupying lesion was found in the liver
03/02/2024	He was admitted to the Department of Gastroenterology of our hospital, and underwent laboratory examination of liver and renal function, serum tumor markers and so on	Abnormal liver function, elevated tumor marker alpha-fetoprotein.
04/02/2024	CT examination of the upper abdomen	Space occupying lesion at the junction of the left and right lobes of the liver, suspected malignant tumor
05/02/2024	MRI examination of the upper abdomen	It is suspected that the liver occupying lesion may be cholangiocarcinoma
07/02/2024	Whole body PET/CT examination	Liver malignancy, no obvious signs of metastasis were found in the rest of the body scan.
13/02/2024	The patient underwent “total hepatectomy and allogeneic orthotopic liver transplantation”	Successful surgery
13/02/2024–19/02/2024	Pathological immunohistochemical examination	Clear cell carcinoma of the liver was diagnosed
21/02/2024	CT examination of the upper abdomen	Liver blood flow is abundant, indicating the success of liver transplantation.
22/02/2024	Patient discharge	Patient discharge
26/03/2024	Telephone follow-up	The patient did not report any discomfort

## Discussion

As is well known, the common pathological type of liver cancer is not otherwise specified hepatocellular carcinoma (NOS-HCC), while clear cell carcinoma is a relatively rare subtype of HCC. Like NOS-HCC, PCCCL also tend to prefer middle-aged and elderly people, and most patients have a history of hepatitis B or hepatitis C, cirrhosis, and can be accompanied by increased AFP (2). Our patient was a 60-year-old woman with elevated serum AFP, consistent with the clinical profile of PCCCL. However, she had not been checked for chronic liver disease before, and the imaging examination of this time indicated cirrhosis, revealing that the patient may have long-term chronic liver disease such as hepatitis B or C, but she did not know it.

Imaging examinations play a significant role in the diagnosis and differential diagnosis of liver tumor lesions. In clinical practice, due to the low incidence rate of PCCCL, there are relatively few research reports on its imaging characteristics. On CT, PCCCL mostly presents low-density shadows, and the higher the proportion of transparent cells, the lower the density of the tumor (6). On contrast-enhanced scanning, the lesion showed uneven enhancement and mainly peripheral enhancement during the arterial phase, and decreased enhancement in the portal vein stage and delayed stage (7). However, there are also a few cases of rapid reinforcement in the arterial phase, rapid decline in the portal phase of the “fast in and fast out” sign (8). The diversity of enhancement modes may be related to the content of clear cells: the higher the content of clear cells, the more inconsistent the enhancement mode is with NOS-HCC; on the contrary, the lower the content of clear cells, the more inclined the enhancement mode is to the “fast in and fast out” enhancement formula of typical hepatocellular carcinoma (9). On MRI, PCCCL mostly showed equal or slightly lower signals on T1WI sequences, and mixed high or slightly higher signals on T2WI sequences. Compared with CT, MRI is more sensitive to reveal the fat components in tumors, and on T2WI sequences, patchy or nodular signal reduction can be seen in lesions, which is one of its characteristic findings, but the probability of occurrence is low (6, 9). Our case showed a slightly low-density shadow on CT, and the contrast-enhanced scan showed rapid enhancement at the edge of the lesion in the arterial phase. With the passage of time, the enhancement degree of the lesion gradually decreased, which was different from the enhancement pattern of most PCCCL reported in the above literature. As for MRI findings, our case was consistent with literature reports, that is, the lesion showed equal signal on T1WI sequence and uneven slightly higher signal on T2WI sequence. To our knowledge, there have been few studies reported on the PET/CT findings of primary PCCCL. The pathologic properties of PCCCL are the same as those of clear cell carcinoma of kidney, ovary and adrenal gland, so it is presumed that its PET/CT findings are consistent with theirs, and it may also be presented by high uptake of ^18^F-FDG, which is reflected in our case, but this needs to be verified in a large number of cases in the future.

PCCCL, as a special subtype of HCC, needs to be differntiated from other subtypes of HCC such as NOS-HCC, macrotrabecular massive subtype HCC (MMS-HCC), steatohepatitic subtype HCC (SHS-HCC), and fibrolamellar subtype HCC (FS-HCC). As the most common subtype of HCC, NOS-HCC showed non-rim arterial phase hyperenhancement and rapid regression in the portal venous phase, presenting a “fast in and fast out” enhancement pattern (10). MMS-HCC is more prone to macrovascular invasion and significant elevation of serum AFP, and the volume of the tumor is usually larger, with a length diameter usually greater than 5.0 cm (11, 12). Steatosis occurs in about 80% of SHS-HCC and is typically characterized by low signal on the inverse-phase T1WI of MRI, and the proportion of steatosis is far more than that of PCCCL (11, 13). For FS-HCC, there are no specific imaging features, but it is more common in younger patients and tends to favor women (14). Moreover, PCCCL also needs to be differentiated from other non HCC diseases including intrahepatic cholangiocarcinoma (IHC), hepatic perivascular epithelioid cell tumors, hepatic hemangioma, etc. IHC on contrast-enhanced CT or T1WI presents mainly peripheral or compartmented enhancement, similar to the enhancement pattern in our patient, but IHC is usually accompanied by adjacent hepatic capsule atrophy and intrahepatic bile duct dilatation, which is different from PCCCL (15). Most of the perivascular epithelioid cell tumors in the liver are angiomyolipomas. Hepatic angiomyolipoma (HAL) showed uneven density on CT, including solid components of the tumor with equal density to the liver parenchyma and low density fat components. On PET, the solid components of the tumor can also present increased ^18^F-FDG uptake, but the maximum standard uptake value (SUVmax) is lower than that of PCCCL. Moreover, HAL usually shows a “fast in and fast out” enhancement pattern on contrast-enhanced CT or T1WI (16). Hepatic hemangiomas usually show uniform isodense or slightly low density on CT. On contrast-enhanced CT or T1WI, peripheral nodular enhancement is common in arterial phase, and progressive centripetal filling is common in venous phase and extended phase (17), which can be differentiated from PCCCL.

As with most tumors, the diagnosis of PCCCL is determined by histopathological examination. Under the microscope, most of the tumor tissues were clear cells with abundant and bright cytoplasm, and the nucleus was located in the center, which was deeply stained and irregular. Some of the liver cells around the tumor were lipoid degeneration, showing chronic interstitial hepatitis changes (18). Immunohistochemical examination showed that almost all PCCCL were positive for Hep par1, and most of them were positive for CK, but few were positive for AFP (19, 20). The case we reported showed diffuse distribution of clear cells within the tumor tissue under the microscope, and the tumor cells expressed positive Hep par1 and CK, which met the pathological diagnostic criteria of PCCCL.

Surgical radical resection of tumors is the main treatment method for PCCCL, and surgical intervention can improve the prognosis of patients (21). For PCCCL with a diameter less than 5.0 cm, some researchers believe that radiofrequency ablation treatment can be performed; however, radiotherapy is not effective in improving the prognosis of patients (22, 23). PCCCL has a better prognosis than other types of liver cancer, but the 5-year survival rate is still low, only 28.1% (24). Therefore, understanding the imaging features of PCCCL is helpful to obtain accurate diagnosis and intervention treatment at an early stage, so as to improve its prognosis. Studies revealed that the size of the mass greater than 5.0 cm in diameter and the absence of surgical resection were independent prognostic factors for PCCCL (22–24). Due to the large mass size of the patient we reported, the maximum diameter of which exceeded 5.0 cm, and the combination of cirrhosis, after full consideration by clinicians and communication with the patient, she chose to undergo total hepatectomy and artificial liver transplantation. After the operation, the patient has been followed up so far, and she is still alive with free of disease.

## Conclusion

In summary, our case study provides a reference for imaging findings of PCCCL, a relatively rare subtype of HCC. On PET/CT, tumors are usually unevenly low-density, along with increased uptake of ^18^F-FDG. On contrast-enhanced CT or T1WI, significant enhancement can appear around the tumor during the arterial phase, and over time, the degree of enhancement of the tumor gradually decreases. Moreover, because tumor cells contain more glycogen and lipid components, resluting in the tumor shows equal or slightly higher signal on T1WI, with certain specificity. These imaging features may provide insight into PCCCL, a relatively rare subtype of HCC, and thus improve its preoperative diagnostic accuracy.

## Data availability statement

The original contributions presented in the study are included in the article/supplementary material, further inquiries can be directed to the corresponding authors.

## Ethics statement

Written informed consent was obtained from the individual(s) for the publication of any potentially identifiable images or data included in this article.

## Author contributions

XH: Conceptualization, Data curation, Formal analysis, Funding acquisition, Methodology, Writing – original draft. LX: Investigation, Methodology, Project administration, Writing – original draft. WZ: Data curation, Formal analysis, Investigation, Writing – original draft. JC: Investigation, Resources, Supervision, Validation, Writing – review & editing. PW: Conceptualization, Formal analysis, Supervision, Visualization, Writing – review & editing.

## References

[1] Mann SA Saxena R. Differential diagnosis of epithelioid and clear cell tumors in the liver. Semin Diagn Pathol. (2017) 34:183–91. doi: 10.1053/j.semdp.2016.12.014 28109715

[2] Liu Z Ma W Li H Li Q. Clinicopathological and prognostic features of primary clear cell carcinoma of the liver. Hepatol Res. (2008) 38:291–9. doi: 10.1111/j.1872-034X.2007.00264.x, PMID: 17877725

[3] Yang SH Watanabe J Nakashima O Kojiro M. Clinicopathologic study on clear cell hepatocellular carcinoma. Pathol Int. (1996) 46:503–9. doi: 10.1111/j.1440-1827.1996.tb03645.x, PMID: 8870006

[4] Kothadia JP Kaur N Arju R Dakhel M Giashuddin S. Primary clear cell carcinoma of the non-cirrhotic liver presenting as an acute abdomen: a case report and review of the literature. J Gastrointest Cancer. (2017) 48:211–6. doi: 10.1007/s12029-016-9831-7 27194053

[5] Maharajan K Hey H Tham I Thamboo TP Wong A Khan IS. Solitary vertebral metastasis of primary clear cell carcinoma of the liver: a case report and review of literature. J Spine Surg. (2017) 3:287–93. doi: 10.21037/jss.2017.06.06, PMID: 28744515 PMC5506305

[6] Wu J Lu AD Zhang LP Zuo YX Jia YP. Study of clinical outcome and prognosis in pediatric core binding factor-acute myeloid leukemia. Zhonghua Xue Ye Xue Za Zhi. (2019) 40:52–7. doi: 10.3760/cma.j.issn.0253-2727.2019.01.010, PMID: 30704229 PMC7351698

[7] Kokubo R Saito K Shirota N Wakabayashi Y Tsuchida A Nagai T. A case of primary clear cell hepatocellular carcinoma comprised mostly of clear cells. Radiol Case Rep. (2019) 14:1377–81. doi: 10.1016/j.radcr.2019.08.021, PMID: 31695824 PMC6823767

[8] Wang H Tan B Zhao B Gong G Xu Z. CT findings of primary clear cell carcinoma of liver: with analysis of 19 cases and review of the literature. Abdom Imaging. (2014) 39:736–43. doi: 10.1007/s00261-014-0104-2 24549879

[9] Liu QY Li HG Gao M Lin XF Li Y Chen JY. Primary clear cell carcinoma in the liver: CT and MRI findings. World J Gastroenterol. (2011) 17:946–52. doi: 10.3748/wjg.v17.i7.946, PMID: 21412505 PMC3051146

[10] Vande Lune P Abdel Aal AK Klimkowski S Zarzour JG Gunn AJ. Hepatocellular carcinoma: diagnosis, treatment algorithms, and imaging appearance after Transarterial chemoembolization. J Clin Transl Hepatol. (2018) 6:175–88. doi: 10.14218/JCTH.2017.00045, PMID: 29951363 PMC6018317

[11] Auer TA Halskov S Fehrenbach U Nevermann NF Pelzer U Mohr R. Gd-EOB MRI for HCC subtype differentiation in a western population according to the 5(th) edition of the World Health Organization classification. Eur Radiol. (2023) 33:6902–15. doi: 10.1007/s00330-023-09669-y, PMID: 37115216 PMC10511376

[12] Cannella R Dioguardi Burgio M Beaufrère A Trapani L Paradis V Hobeika C. Imaging features of histological subtypes of hepatocellular carcinoma: implication for LI-RADS. JHEP Rep. (2021) 3:100380. doi: 10.1016/j.jhepr.2021.100380, PMID: 34825155 PMC8603197

[13] Bannasch P Ribback S Su Q Mayer D. Clear cell hepatocellular carcinoma: origin, metabolic traits and fate of glycogenotic clear and ground glass cells. Hepatobiliary Pancreat Dis Int. (2017) 16:570–94. doi: 10.1016/S1499-3872(17)60071-7, PMID: 29291777

[14] Eggert T McGlynn KA Duffy A Manns MP Greten TF Altekruse SF. Fibrolamellar hepatocellular carcinoma in the USA, 2000-2010: a detailed report on frequency, treatment and outcome based on the surveillance, and End Results database. United European Gastroenterol J. (2013) 1:351–7. doi: 10.1177/2050640613501507, PMID: 24917983 PMC4040774

[15] Saleh M Virarkar M Bura V Valenzuela R Javadi S Szklaruk J. Intrahepatic cholangiocarcinoma: pathogenesis, current staging, and radiological findings. Abdom Radiol. (2020) 45:3662–80. doi: 10.1007/s00261-020-02559-7, PMID: 32417933

[16] Yang W Sun Q Shang M Li S Hu X Hu X. Multimodal imaging study of hepatic perivascular epithelioid cell tumors: a case report. Front Med. (2023) 10:1322048. doi: 10.3389/fmed.2023.1322048, PMID: 38173942 PMC10762310

[17] Liu Z Yi L Chen J Li R Liang K Chen X. Comparison of the clinical and MRI features of patients with hepatic hemangioma, epithelioid hemangioendothelioma, or angiosarcoma. BMC Med Imaging. (2020) 20:71. doi: 10.1186/s12880-020-00465-4, PMID: 32600273 PMC7322860

[18] Lao XM Zhang YQ Jin X Lin XJ Guo RP Li GH. Primary clear cell carcinoma of liver--clinicopathologic features and surgical results of 18 cases. Hepato-Gastroenterology. (2006) 53:128–32. PMID: 16506391

[19] Murakata LA Ishak KG Nzeako UC. Clear cell carcinoma of the liver: a comparative immunohistochemical study with renal clear cell carcinoma. Mod Pathol. (2000) 13:874–81. doi: 10.1038/modpathol.3880156, PMID: 10955454

[20] Adamek HE Spiethoff A Kaufmann V Jakobs R Riemann JF. Primary clear cell carcinoma of noncirrhotic liver: immunohistochemical discrimination of hepatocellular and cholangiocellular origin. Dig Dis Sci. (1998) 43:33–8. doi: 10.1023/a:1018859617522, PMID: 9508531

[21] Li Z Wu X Bi X Zhang Y Huang Z Lu H. Clinicopathological features and surgical outcomes of four rare subtypes of primary liver carcinoma. Chin J Cancer Res. (2018) 30:364–72. doi: 10.21147/j.issn.1000-9604.2018.03.08, PMID: 30046230 PMC6037584

[22] Ji SP Li Q Dong H. Therapy and prognostic features of primary clear cell carcinoma of the liver. World J Gastroenterol. (2010) 16:764–9. doi: 10.3748/wjg.v16.i6.764, PMID: 20135727 PMC2817067

[23] Wen J Yao X Xue L Aili A Wang J. Predictors and survival of primary clear cell carcinoma of liver: a population-based study of an uncommon primary liver tumor. Transl Cancer Res. (2021) 10:3326–44. doi: 10.21037/tcr-21-9, PMID: 35116639 PMC8798129

[24] Chen ZS Zhu SL Qi LN Li LQ. Long-term survival and prognosis for primary clear cell carcinoma of the liver after hepatectomy. Onco Targets Ther. (2016) 9:4129–35. doi: 10.2147/OTT.S104827, PMID: 27462167 PMC4939989

